# The costs and benefits of deep brain stimulation in Parkinson's disease: a review and social network analysis

**DOI:** 10.1055/s-0045-1809996

**Published:** 2025-07-17

**Authors:** Carlos Zúñiga-Ramírez, Katia Carmina Farías-Moreno, Gabriel Moreno, Enrique Gómez-Figueroa, Hernando Efraín Caicedo-Ortíz, José Damián Carrillo-Ruíz

**Affiliations:** 1 Hospital Civil de Guadalajara “Fray Antonio Alcalde”, Unidad de Movimientos Anormales y Enfermedades Neurodegenerativas, Guadalajara, Mexico.; 2 University of Central Florida-Hospital Corporation of America Florida Healthcare, Department of Neurology, Orlando FL, United States.; 3 Hospital Civil de Guadalajara “Fray Antonio Alcalde”, Departamento de Neurología, Guadalajara, Mexico.; 4 Universidad Anahuac, Ciudad de México, Mexico.; 5 Universidad Autónoma de la Ciudad de México, Ciudad de México, Mexico.; 6 Universidad Anahuac, Facultad de Psicología, Coordinación de Neurociencias, Ciudad de México, Mexico.; 7 Hospital General de México, Departamento de Neurocirugía Estereotáctica y Funcional, Ciudad de México, Mexico.

**Keywords:** Parkinson Disease, Levodopa, Deep Brain Stimulation, Cost-Effectiveness Analysis, Cost-Benefit Analysis

## Abstract

**Background:**

Parkinson's disease (PD) is the second most prevalent neurodegenerative disorder worldwide. Levodopa has been considered the best treatment option. However, deep brain stimulation (DBS) use has increased over time, mostly when levodopa-related complications arise.

**Objective:**

To review the current evidence regarding economic evaluations assessing costs and benefits comparing pharmacological versus surgical treatment among subjects with PD.

**Methods:**

We searched three databases (PubMed, Embase, and Google Scholar) for studies comparing levodopa treatment and DBS among subjects with PD in terms of costs and benefits from therapy.

**Results:**

Out of the 107 studies identified, 14 met the inclusion criteria. Most of the published studies were from Europe. Incremental cost-effectiveness ratios have shown variable results, from -€979 to €6,729 per change of 1 point in the score on part III of the Unified Parkinson's Disease Rating Scale (UPDRS III), while incremental cost-utility ratios depict values as low as €6,700 and as high as $704,906.03 per quality-adjusted life-years (QALY).

**Conclusion:**

We observed a higher cost during the 1 ^st^ year of DBS implantation due to the surgical procedure itself, subsequently, there was a trend for a lower cost over the following years, with no loss of benefit. Overall, the studies showed DBS as a cost-effective measure at 5-years after implantation.

## INTRODUCTION

Parkinson's disease (PD) is the second most prevalent neurodegenerative diseases worldwide. The number of affected people is constantly increasing: crude prevalence grew by 74% and global age-standardized prevalence rates increased by 21.7% from 1990 to 2016. Furthermore, deaths and disability-adjusted life-years (DALYs) also grew to around 20% during that time, 1 while projected data estimate that 2.6 million cases in 1990 will expand to more than 17 million by 2040. 2

Global PD prevalence and incidence have been reported as high as 440.3 cases per 100,000 people, and 36.6 cases per 100,000 people/year, respectively. 3 A recent meta-analysis estimated the prevalence and incidence of PD in Latin America, depicting 472 per 100,000 and 31 per 100,000 people/year, respectively. 4

Currently, no cure exists for PD. Therefore, it evolves into a chronic disease, increasing costs of medical attention and decreasing quality of life (QoL) over time. Levodopa has been the mainstay of treatment, 5 but as PD advances over time, the presence of motor fluctuations and levodopa-induced dyskinesias become more frequent, increasing treatment costs. Although different therapies have been employed to improve these conditions, none of them have been resolutive.

Deep brain stimulation (DBS) of the subthalamic nucleus (STN) was introduced during the 1990s, proving to be an excellent alternative for advanced PD treatment. 6 Although it is effective in treating advancing-disease complications, the costs associated with this therapy are high. There still exists uncertainty regarding treatment costs and efficacy over time in people suffering from PD, with some advocating for medical treatment while others promoting surgery as the best option.

The present study aim to review the current evidence regarding economic evaluations assessing the cost-benefit of pharmacological and DBS treatment among subjects with PD.

## METHODS

**Figure 1:**
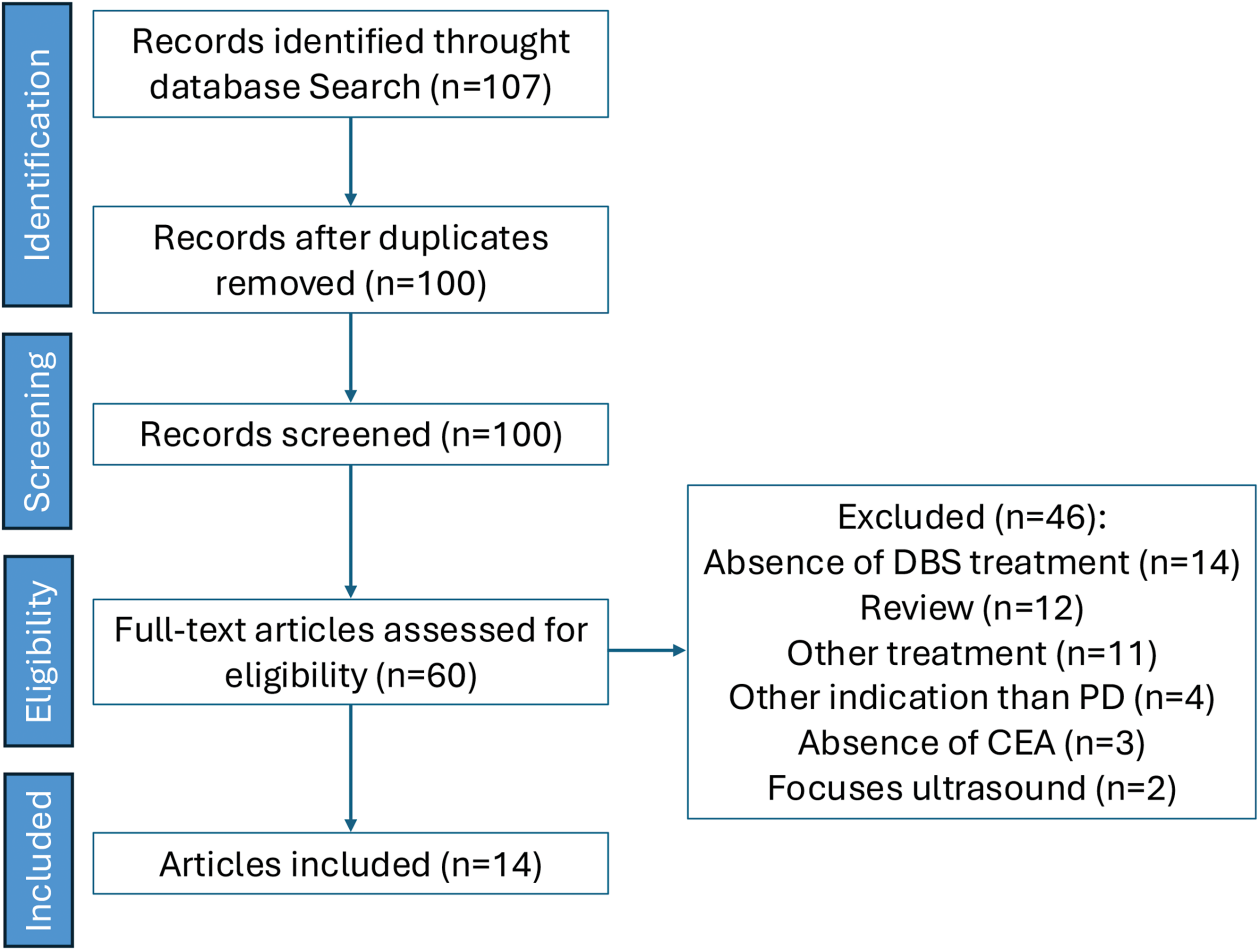
Process of selection of included studies. **Abbreviation:** CEA, cost-effectiveness analysis.

Two independent authors searched the PubMed, Embase, and Google Scholar databases from their inception up to November 15 ^th^ , 2024, for articles with the following MeSH terms: Parkinson's Disease AND economic evaluations, cost-effectiveness, cost-benefit, cost-utility, and cost-minimization. We included the studies comparing medical treatment (oral levodopa and other oral medications) and DBS among subjects with PD. A manual search of the references of the articles initially found was also performed. There was no language restriction. We excluded systematic reviews and national clinical guidelines from this analysis, but these types of papers were manually reviewed to assess the completeness of the studies retrieved by the literature search. Studies in which PD was not the only indication for DBS were also excluded from analysis. Studies comparing DBS with therapies different from oral medications, such as intrajejunal levodopa or focused ultrasound, were not included in the review. Discrepancies were solved by a third reviewer. The study selection process is shown in Figure 1 .

Two independent authors collected the included studies which were sorted by: author, year, country, study type (cost-utility or -effectiveness analysis), model (Markov, semi-Markov, prospective or retrospective), subject characteristics (early or advanced PD), intervention, perspective, time horizon, discount rate, incremental cost-effectiveness ratio/incremental cost-utility ratio (ICER/ICUR) per quality-adjusted life-years (QALY), and costs (direct or indirect). Discrepancies were solved by a third reviewer.

Not all studies presented the value of their currency according to the year, so the monetary value was assumed according to the date of publication. Currency values not reported in US dollars were converted according to the study year and dollar conversion rate for that specific time.

Finally, a social network analysis was performed using Gephi (University of Technology of Compiègne), version 0.10, which assessed the relationship of published articles between countries, as well as the association between costs and obtained benefits in regards of ICER/ICUR among the different publications. The Fruchterman & Reingold algorithm was employed to perform a physical simulation to find a node arrangement that minimizes overlaps and distributes nodes in a balanced way. 7 The ICER/ICUR values were standardized to measure the correlation strength between studies and outcomes, presented in a 0 to 100 scale, with 0 as the weakest correlation and 100 the strongest. The node size is determined by the number of publications assessing that particular outcome.

## RESULTS

We retrieved 14 articles that performed an economic evaluation between DBS and medical therapy among subjects with PD. The study features are summarized in Table 1 . 7 8 9 10 11 12 13 14 15 16 17 18 19 20 21 Social network analysis found a very low density (2.4%) and high modularity (56.9%), meaning that there is a dense connection between authors publishing this type of study, but not between authors from different countries.

**Table 1:** Main features of the included studies

#	Author (year)		Country	Study type	Model	Subjects	Intervention (n)	Perspective	Time horizon	Discount rate	Currency value	ICER/ICUR	Converted ICER/ICUR to US dollars	Costs
1	Tomaszewski and Holloway (2001) 8		USA	Cost-utility	Semi-Markov	H&Y 3–5 ≥50 yo	STN/GPi DBS vs BMT	Societal	Lifetime	3%	ND	$49,194/QALY	–	Direct
2	Spottke et al. (2002) 9		Germany	Cost-effectiveness	Prospective	Advanced PD	DBS STN (16)	Health care	1 year	5%	November 1999	DM28,580/UPDRS I point DM7,935/UPDRS II point DM6,440/UPDRS III point DM4,170/UPDRS IV point DM1,800/total UPDRS point	$14,860/UPDRS I point $4,126.2/UPDRS II point $3,348.8/ $2,170/UPDRS IV point $940/total UPDRS point	Direct
3	Meissner et al. (2005) 10		Germany	Cost-effectiveness	Retrospective	Advanced PD	DBS STN (46)	Health care	3 years (one before, 2 after DBS)	5%	February 2004	€979/UPDRS III point	$1,215.72/UPDRS III point	Direct
4	Valldeoriola et al. (2007) 11		Spain	Cost-effectiveness Cost-utility	Prospective	Advanced PD	STN DBS (14) vs BMT (15)	Patient and health care	1 year	ND	ND	€239.8/total UPDRS point €34,389/QALY	$328.64/total UPDRS point $47,129.44/QALY	Direct
5	Dams et al. (2013) 12		Germany	Cost-effectiveness Cost-utility	Markov	60 yo advanced PD in off state H&Y 3: 50% H&Y 4: 30% H&Y 5: 20%	DBS vs medical treatment	Health care	Lifetime	3%	2010	€6,700/QALY 1 year: €15,214/UPDRS II point €6,729/UPDRS III point 2 years: €14,264/UPDRS II point €3,539/UPDRS III point 5 years: €13,086/UPDRS II point €3,253/UPDRS III point 10 years: €9,800/UPDRS II point €2,456/UPDRS III point	$8,882.32/QALY year: $16,192.34/UPDRS II point $8,920.77/UPDRS III point 2 years: $18,910.1/UPDRS II point $4,691.72/UPDRS III point 5 years: $17,348.37/UPDRS II point $4,312.57/UPDRS III point 10 years: $12,992.1/UPDRS II point $3,255.97/UPDRS III point	Direct
6	Eggington et al. (2014) 13		UK	Cost-utility	Markov	Advanced PD	DBS + BMT vs BMT (156)	Payer	5 years	3.5%	2011	£20,678/QALY	$33,268.83/QALY	Direct
7	Zhu et al. (2014) 14		Hong Kong	Cost-effectiveness	Prospective	Advanced PD	STN DBS (13)	ND	2 years	3%	2010	1 year: $926/UPDRS III point 2 years: $421/UPDRS III point 1 year: $123,110/EQ-5D point 2 years: $62,846/EQ-5D point	–	Direct
8	McIntosh et al. (2016) 15		UK	Cost-utility	Prospective	PD subjects (ND)	STN/GPi DBS (183) vs BMT (183)	Health and social care	10 years	3.5%	June 2010	1 year: £468,528/QALY 5 years: £45,180/QALY 10 years: £70,537/QALY	year: $700,402.5/QALY 5 years: $67,539.58/QALY 10 years: $105,445.76/QALY	Direct
9	Dams et al. (2016) 16		Germany	Cost-effectiveness Cost-utility	Markov	52 ± 6.6 yo PD subjects H&Y 1: 5% H&Y 2: 65% H&Y 3: 20% H&Y 4: 10% Motor complications ≤ 3y Disease duration ≥ 4y	DBS vs medical treatment	Health care	lifetime	3%	2013	€22,710/QALY €89/PDQ39 point	$30,161.6/QALY $118.2/PDQ39 point	Direct
10	Fundament et al. (2016) 17		UK	Cost-utility	Markov	Early PD subjects	DBS + BMT vs BMT	Health care	15 years	3.5%	ND	£19,887/QALY	$26,954.84/QALY	ND
11	Pietzsch et al. (2016) 18		USA	Cost-utility	Markov	Advanced PD	DBS + BMT vs BMT	Health care	10 years	3%	2014	$23,404/QALY	–	Direct
12	Kawamoto et al. (2016) 19		Japan	Cost-utility	Markov	60 yo male Early and advanced PD	DBS vs medical treatment	Health care	10 years	ND	April 2014	Early: $70,200/QALY Intermediate: $27,200/QALY Late: $27,000/QALY	–	Direct
13	Fann et al. (2020) 20		Taiwan	Cost-effectiveness Cost-utility	Markov	Advanced PD	DBS vs medical treatment	Societal	10 years	3%	ND	3 years: $147,065/LYG $123,436/QALY 10 years: $36,883/LYG $69,033/QALY	–	Direct
14	Guo et al. (2023) 21		China	Cost-utility	Markov	Advanced PD	DBS vs BMT	Patient	15 years	ND	ND	1 year: $704,906.03/QALY 15 years: $32,549.96/QALY	–	Direct

Abbreviations: BMT, best medical treatment; EQ-5D, European Quality of Life-5 Dimensions; DBS, deep brain stimulation; GPi, internal globus pallidum; H&Y, Hoehn & Yahr scale; ICER/ICUR, incremental cost-effectiveness ratio/incremental cost-utility; ND, not declared; QALY, quality-adjusted life-years; STN, subthalamic nucleus; UPDRS, Unified Parkinson's Disease Rating Scale.

**Figure 2:**
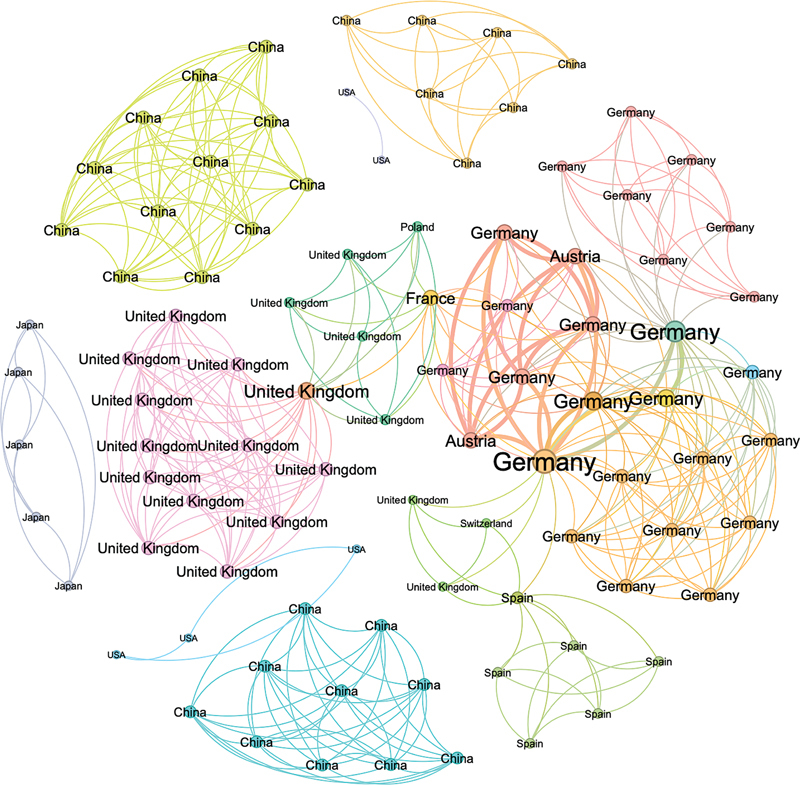
Social network analysis showing the correlations between different countries and performed studies.

Cooperative studies were commonly seen amongst the same authors from European countries. However, groups from Asia, United States, and Canada tended to publish independently. Germany published the most studies, however, cooperation between European countries was frequently seen, establishing a link between Germany and the United Kingdom through France. Countries from Asia have their own cooperative study networks, often unrelated even to other Asian countries; however, a cooperative network between USA and China was also seen ( Figure 2 ). Many of the evaluated subjects were treated with STN DBS.

In 2001, Tomaszewski and Holloway 8 assessed the ICUR of DBS in subjects with advanced PD using a semi-Markov model. This study included STN or pallidal (GPi, internal globus pallidum) DBS in subjects aged 50 years and older with a Hoehn & Yahr (H&Y) stage of 3 to 5 suffering from severe motor fluctuations. Baseline utility estimates for establishing QALY were derived from a previous cross-sectional study.

The costs of surgical therapy were estimated using a combination of professional opinions, Medicare billing, and pricing from the Strong Memorial Hospital in Rochester, New York. A discount rate of 3% has been applied for future QALY. Complications from DBS accounted for a 20% decrease in it as well. Although the currency values are not specified by year, the costs for medications are reported as from 2000. 8

Analysis was performed from the societal perspective and time horizon was the remaining life expectancy of the subject. They found a QALY of 7.08 and 7.8 for medical and surgical therapy, respectively. The costs of medical treatment were $417,000, compared with $452,000 for surgery, yielding an ICUR of $49,194 per QALY. The authors concluded that DBS would be more cost-effective for treating advanced disease over time. However, due to the study design, results are not accurate for a real-world situation. 8 In the present analysis, the targets were not analyzed separately despite their unique effects on PD subjects, such as a reduction in dopaminergic medication intake seen in STN targeting.

Spottke et al. 9 published the costs and effectiveness of STN DBS, performed in 16 subjects with advanced PD followed prospectively for 1 year from two centers in Germany. The main measure of effectiveness was through the Unified Parkinson's Disease Rating scale (UPDRS) which is composed of four parts:

- cognitive/behavioral;
- activities of daily living;
- motor skills, and
- motor complications from therapy.

Increase in score means worsening of condition. Direct costs of therapies were obtained from the data of the statutory health insurance in Germany, a health care perspective was adopted, and a 5% deduction rate was applied. Monetary costs were expressed in Deutsche Marks (DM) from November 1999 (1 DM = $0.52 = €0.51 = £0.32). The authors found an ICER at 1 year of DM28,580 ($14,860; €14,580; £9,150) per 1-point improvement in the score on part I of the UPDRS, of DM 7,935 ($4,126.2; €4,046.85; £2,539.2) in the score on part II of the UPDRS, of DM6,440 ($3,348.8; €3,284.4; £2,060.8) in the score on part III of the UPDRS, of DM4,170 ($2,170; €2,130; £1,330) in the score on part IV of the UPDRS IV, and of DM 1,800 ($940; €918; £576) per 1-point improvement in the total UPDRS score. It is noteworthy that only one of 16 studied subjects got employment after the surgical procedure. The assessed population and length of time employed undermine the obtained results. 9

A multicenter study 10 evaluated costs and effectiveness of STN DBS among 46 advanced PD subjects (mean age at time of DBS implantation: 58.6 ± 1.0 years, mean disease duration: 16.0 ± 0.7 years) from two German centers. Costs and motor skills were assessed through a cost-effectiveness analysis, including directly from a health care perspective and exerted a 5% discount rate. The exchange rate employed was from February 2004 (1 € = $1.24, £0.67).

Subjects were analyzed in a retrospective fashion: 1 year before surgery and 2 years after it. Costs for medical treatment before surgery were of €11,230 which decreased to €3766 for the first year and €4,449 for the second year after DBS. Costs derived from admissions to the hospital were also reported: €4,676 before DBS, €17,231 at 1 year, and €2,689 at 2 years after DBS. Total expenses for each subject were also recorded, €15,991 before surgery, €21,082 at 1 year, and €7,223 at 2 years after DBS. It is noteworthy to mention that none of the subjects included returned to work once DBS was performed. The UPDRS III scores were 18.5, 13.3, and 14.5 when tested before, and at 1 and 2 years after DBS, respectively. 10

With data regarding costs and improvements of 1 point in the UPDRS III score, an ICER of -€979 was obtained for the first year after surgery only since expenses and scores were significantly lower than preoperatory results. The cost increase during the first year is attributed to the surgical procedure itself. Thereafter, there is a trend toward lower costs over subsequent years without a loss of benefit from DBS. 10

In 2007, a longitudinal study from Spain 11 evaluated the best medical treatment (BMT) and STN DBS among subjects with advanced PD with severe disability. Assessment of cost, effectiveness, and health-related QoL (HRQoL) were the main endpoints of this study. There were 14 subjects with STN DBS compared with 15 subjects under BMT for one year. Direct costs (both medical and non-medical) were only calculated, obtained from a Spanish research center database (SOIKOS), however, neither discount rate nor currency exchange rate were described. The European Quality of Life-5 Dimensions (EQ-5D) was employed to calculate QALY. The obtained ICUR was €34,389 per QALY, while the ICER consisted of €239.8 per one point improvement at the total UPDRS score. Time horizon and number of assessed subjects are some of the limitations of this study. 11

Dams et al. 12 in 2013 developed a Markov model to assess cost-effectiveness of DBS compared with medical treatment in Germany. It included 6 states: H&Y I to V and death as the sixth state. A lifetime horizon was proposed for the model, changing cycles every year. Subjects were around 60-years-old with advanced PD experiencing motor fluctuations and dyskinesias. Death was defined using mortality ratios from 2009 in Germany.

Direct costs were analyzed in this model. Utilities included the EQ-5D score and QALY, with an annual discount rate of 3% for the latter. The values obtained for each score also came from multivariate analysis and expert opinion. A threshold below 50,000 Canadian dollars (approximately € 31,645) per QALY was considered a cost-effective strategy. Cost data were customized from 2010 rates. 12

Mean discounted lifetime costs for medical treatment were €126,200 compared with €133,200 for those who underwent surgical treatment. Among subjects medically treated, calculated QALY was 10.6, while DBS improved it to 11.6. The resultant ICUR was €6,700 per QALY. Estimated lifetime ICUR depicted €6,677 per QALY while calculated ICER at 1, 2, 5, and 10 years for UPDRS II were of €15,214, €14,264, €13,086, and €9,800, respectively. Regarding the computed ICER for UPDRS III at 1, 2, 5, and 10 years, results were €6,729, 3,539, 3,253, and 2,456 each. The hypothetical nature of design is the mayor disadvantage of the study. 12

In 2014, a Markov model was performed by Eggington et al. 13 assessing the cost-utility of DBS plus BMT versus only BMT among subjects with advanced PD. A payer's perspective was used in the model, which collected data from a randomized controlled trial that included 156 subjects with advanced PD. A time horizon of 5 years was proposed, and subjects' health was according to H&Y classification during the off phase, which also was subdivided into 4 stages, depending upon the time spent in off. An annual discount rate of 3.5% was applied while costs were from the year 2011. Utilities were calculated through 2 different economic evaluations, which were previously published. Authors found that DBS plus BMT were cost-effective, as £20,678 per QALY were estimated from the study. Also, 13.7% of subjects under DBS and 17.2% of those with medical treatment died, according to the model. The theoretical design of the study, as well as the incorporation of data from other different trials was a limitation of this study. 13

Zhu et al. 14 reported a cost-effectiveness analysis from 13 subjects with disabling PD that underwent for DBS treatment and were followed for 2 years. Follow up measures included change in the UPDRS III and the EQ-5D scores at 1- and 2-years postimplantation. A discount rate of 3% was applied during the second year. The exchange rate was from 2010. The baseline cost per subject was $4,186, this sum increased to $29,178 during the first year and decreased to $1,490 in the second year. The ICER regarding improvement of the UPDRS III was $926 during the first year and $421 during the second. Relative to EQ-5D, the ICER was $123,110 for the first year and $62,846 for the second. The small number of participants in the study, as well as the lack of information concerning design perspective are some of its main limitations. 14

McIntosh et al. 15 assessed the costs and outcomes of DBS and BMT at 1-year and predicted the economic behavior from both therapies up to 10 years. All data was collected from a previous trial (PD-SURG) and questionnaires were applied to PD subjects who underwent either medical or surgical therapy. The discount rate was settled at 3.5%. The pricing was from June 2010.

Missing data was imputed. The EQ-5D score was employed to calculate utilities from baseline to 1 year. Next, combining the latter with survival data retrieved from PD-SURG, the QALY were estimated. After this, an ICER was calculated, and a deterministic extrapolation and a cost-effectiveness model were generated to estimate costs at 5 and 10 years. Costs of 183 subjects on each arm were obtained in 1year, resulting in an ICER of £468,528 per QALY, concluding that surgery is less likely to be cost-effective at 1 year. At 5 years, extrapolating costs from surgery and BMT, an ICER of £45,180 per QALY was estimated, favoring surgery over BMT. At 10 years, the calculated ICUR was of £70,537 per QALY, raising questions regarding cost-effectiveness of DBS therapy. Most of this change has to do with battery replacement costs. Currently, battery life may be 15 years, roughly 10 years longer than it had been in the 2010's, which certainly would affect the results. 15

In 2016, four new Markov analysis were published, two of them focusing on younger PD subjects receiving either medical treatment or STN DBS. In the first model, 16 direct costs were considered from a healthcare perspective and subjects' age ranged between 18 and 60 years. The H&Y stage was from I to IV in off state and motor complications were not present for more than 3 years. A lifetime time horizon was done. The disease duration was at least 4 years. Utilities were measured using EQ-5D score, while effectiveness was estimated using the 39-Item Parkinson's Disease Questionnaire (PDQ-39) summary index. Costs were adapted from 2013 and a discount rate of 3% was employed. Subjects' features were adopted from the EARLYSTIM study. The surgical procedure costs were €31,000, and battery replacement every 5 years had a cost of €15,000. Decrease in QoL was contemplated during the first 3 months after DBS surgery. An ICUR of €22,710 per QALY, and an ICER of €89 per PDQ-39 point were obtained. 16

The second Markov model assessed from a UK health care perspective, the costs and QALY of BMT versus BMT plus DBS. 17 This model used a time horizon of 15 years and an annual discount rate of 3.5%. Clinical data was retrieved from the EARLYSTIM study, as well as a systematic literature review performed in PubMed, Cochrane, and Embase. Mortality data was applied from UK all-cause mortality rates. Cost data was obtained from price lists, hospital billing, and social care data. The QoL was obtained from measures employed at the EARLYSTIM study. Analysis revealed that DBS compared with BMT obtained an ICUR of £19,887 per QALY. Cost data regarding the year for which it was obtained is not described.

The third model evaluated advanced PD subjects from 2 simulated cohorts, one of which consisted of subjects treated with DBS and BMT and the other where only BMT was used, from a US health care perspective. 18 The analysis was done using a 10-year time horizon, with an annual discount rate of 3%, and cost values from 2014. Most of the data was retrieved from a randomized controlled trial published in 2006. Direct costs were calculated based on Medicare's claims forms regarding either PD medications or DBS. The QoL was collected from previous studies about PD. After analysis for a 10-year period, the authors estimated an ICUR of $23,404 per QALY, favoring surgical treatment.

Lastly, from a Japan health care perspective, a Markov model was performed comparing cost-effectiveness of DBS and medical therapy among male subjects with either early or late-stage PD using a 10-year time horizon. 19 Cost rates were calculated from April 2014. Motor outcomes were estimated using H&Y stages found in a separate Japanese study. Since no QoL studies in Japanese population were published, a survey using EQ-5D was performed by the authors on 62 healthy volunteers to collect this information. The calculated ICUR in early, intermediate, and late-stage PD were $70,200, 27,200, and 27,000 per QALY respectively.

In 2020, a new Markov model 20 simulated 10 thousand subjects with advanced PD followed for 3 and 10 years after STN DBS, based on societal perspective. Both cost-utility and cost-effectiveness were calculated with an annual 3% discount rate. The exchange rate year is not described. Clinical features were adopted from another study while multivariate analysis was performed to generate each subject's motor score. The DBS costs were obtained from a medical center and from the Bureau of National Health Insurance of Taiwan. Life years gained (LYG) were used as a measure of effectiveness, while QALY was used for utilities. Information for modelling these measures was obtained from four other studies. The 3-year ICER was $147,065 per LYG, and the ICUR was $123,436 per QALY. At 10 years, $36,883 per LYG and $69,033 per QALY were calculated as the ICER and ICUR, respectively. It turns out that STN DBS was cost-effective in this nation because costs did not surpass the Gross Domestic Product more than 3-fold. 20

In 2023, Guo et al. 21 performed a cost-utility analysis from clinical data and costs obtained retrospectively from 2014 to 2020 in China. Included subjects had advanced stage PD, who were treated with DBS. A Markov simulation model was employed to determine costs and utilities from a 15-year time horizon. Patient payer perspective was considered in this study. The QoL was obtained from 2 previous studies with PD subjects, while transition probabilities were adopted from expected changes in H&Y scale. Costs of DBS were calculated from China Healthcare Security's data.

Although costs were obtained from 2014 to 2020, currency values are not standardized to any particular year. The costs for BMT were $7,439.68, while those for DBS were $56,515.2. The QALY gained from BMT and DBS were 0.3885 and 1.8962, respectively. The estimated ICUR during the first and 15 ^th^ year were $704,906.03 and $32,549.96 per QALY, respectively. The net benefit was also calculated, with $5,154.94 for BMT and $4,627.85 for DBS at 15 years. This measure remained negative for DBS during the first 5.5 years, after that it increased to positive values, while BMT showed a positive net benefit along the studied time horizon. Although sensitivity analysis mentions a discount rate of 0%, the latter was not specified at original calculations. This paper demonstrates the costs and benefits obtained from a developing country's perspective. However, the information obtained comes from a simulation model. Additionally, no details were given regarding which DBS target was elected. 21

**Figure 3:**
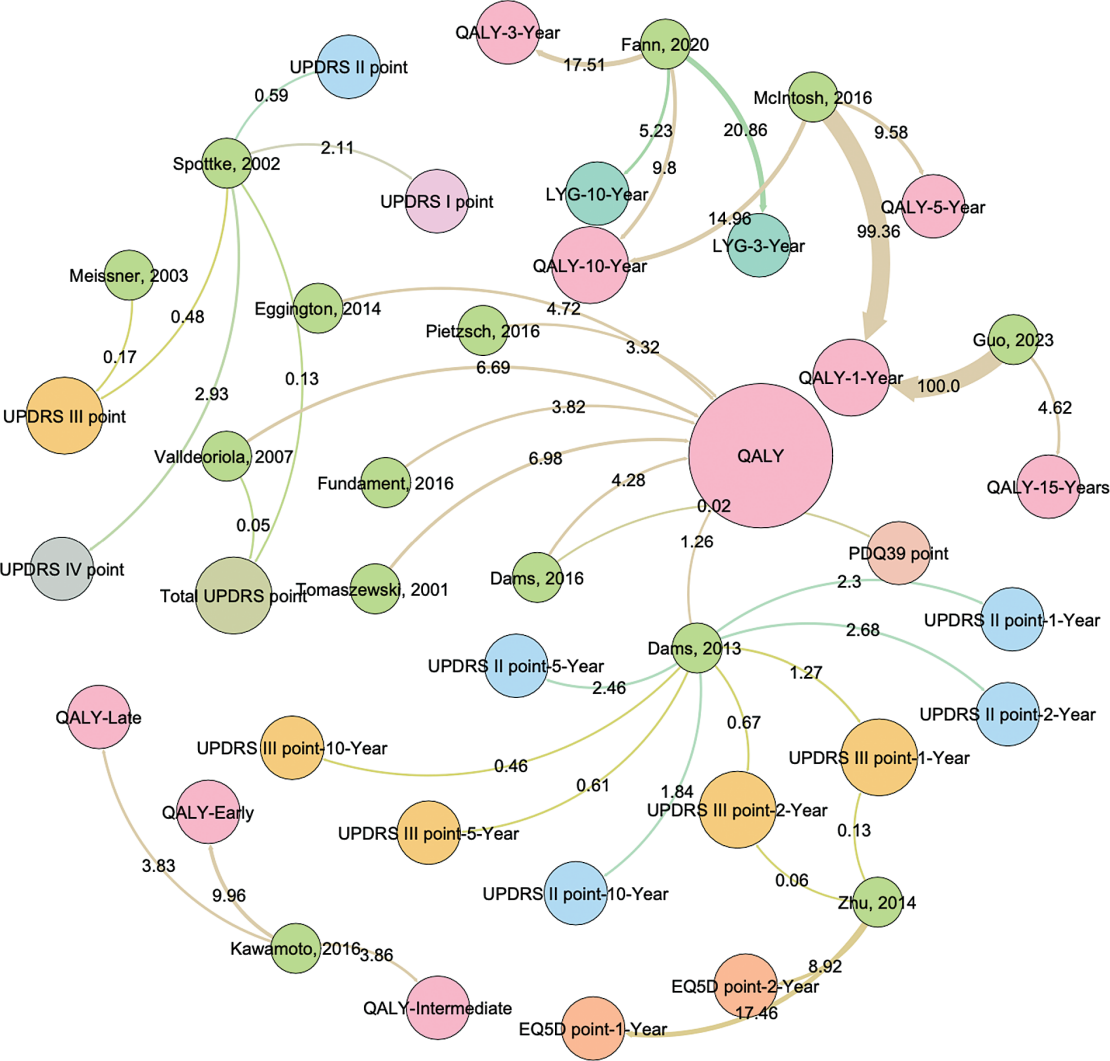
Abbreviations: EQ-5D, European Quality of Life-5 Dimensions; LYG, life-years gained; PDQ39, 39-Item Parkinson's Disease Questionnaire; UPDRS, Unified Parkinson's Disease Rating Scale. Social network analysis depicting the correlations between the article's incremental cost-effectiveness ratio/incremental cost-utility ratio (ICER/ICUR) and the outcome employed. Numbers represent the correlation (from 0 to 100) of ICER/ICUR standardized values between studies and outcomes. Line width represents correlation strength. Node size represents the number of studies involved in that particular outcome.

A bipartite unidirectional social network analysis between study outcomes and ICUR/ICER was performed. This found that studies from Guo et al. 21 and McIntosh et al. 15 have the strongest correlation between QALY at 1 year and ICUR/ICER, with 100% ($704,906.03 per QALY) and 99.36% ($700,402.5 per QALY), respectively. This means that the highest values regarding cost and utilities at 1-year of surgical treatment are better represented by these studies. The articles showing the weakest correlation between utilities and ICER/ICUR are Valldeoriola et al., 11 Zhu et al., 14 and Dams et al., 16 with the first showing a correlation of 0.05 per total UPDRS point, the second one of 0.06 per UPDRS III point at 2 years, and the third study of 0.02 per QALY ( Figure 3 ). The lowest percentage correlation between the study and any particular outcome suggests that additional discrepancies will be found between the ICER/ICUR described in that particular article.

## DISCUSSION

Parkinson's disease has been recognized as a disabling disease, not only by the motor derangement it produces to affected individuals, but also due to its effects on general welfare. Previous reports considered the exerted effects of disease as a “premature aging,” since employment, social life and leisure activities were highly compromised among affected subjects. 22

Treatment with DBS opened a new paradigm regarding PD. Improvement of cardinal features are seen during acute and chronic states of disease and decrease of total medication regimen is usually achieved. 23 24 25 However, its high surgical cost and invasive nature makes the procedure not suitable for all cases. Heterogeneous results were found in the current literature, since each study used different currency, time horizons, and measures of benefit. Furthermore, only 10 out of 14 studies calculated QALY, based on prior QoL assays. Nonetheless, surgical treatment with DBS seems to become more a cost-effective intervention over time, when used as the core therapy.

Concerns regarding the real costs of PD treatment had been known for long time. 26 27 28 Work absenteeism, social isolation, and medical expenditures are some of the main issues this cohort must deal with, showing mean health expenses of $10,168, compared with 4,743 for those without PD in the late 1990s. 27 Loss of earnings and informal caregiving produced the most impact from a societal perspective, accounting for more than $25,000 annually (price listing from 1994, in US dollars). 28 More recent data in United States show that total economic burden in 2017 was of $51.9 billion, with $25.4 billion corresponding to direct medical costs, while $26.5 billion belonged to indirect medical costs. Moreover, this economic burden will increase to $79.1 billion by year 2037, as projections expect more than 1.6 million PD cases by that time. 29

Economic evaluations in PD were introduced in the late 1990s. The first assessment of this nature was performed by Hoerger et al. in 1998 comparing the introduction of pramipexole among disease stages. 30 They observed that adding pramipexole to early-stage subjects with PD resulted in a cost of $8,837 per QALY, while an increment of $12,294 per QALY was found when it was added in later stages. Since levodopa is the cornerstone of medical treatment among PD subjects, all the assessed studies compared levodopa against DBS. Besides this, current concerns have made dopamine agonists fall into disuse due to secondary effects. 31

Heterogeneous results were obtained from the included studies. ICER showed different values, from -€979 to €6,729 per change in one point in the UPDRS III score, while incremental cost-utility ratios varied from $6,700 to 704,906.03 per QALY. Currency conversion values, discount rate, time horizon, perspective, and study type are the main causes of heterogeneity across the studies.

There were nine studies that employed a Markov model for cost and benefit estimation over time. The main advantage using this type of modelling relies on predicting disease behavior at the long run, simulating the progress and evolution of chronic diseases. Besides this, the cost of research is greatly reduced by this type of modelling compared with expensive prospective studies. Although Markov models will provide an accurate approximation to reality, the results are simulated, and illness behavior itself tends not to be so predictable in nature. The latter must be considered mainly among studies based upon a lifetime horizon. Besides this, the probability of transition from one state to another is based merely on the subjects' current state, not considering prior states affecting progression. The latter is also known as the “Markovian assumption” and is one of the main issues regarding simulation models. 32

Out of the 14 available studies, 7 come from Europe, more specifically from Germany 9 10 12 16 and the United Kingdom. 13 15 17 The costs and subjects' perspectives are quite different from those of developing countries, due to cultural perception of health and wellness, as well as derived costs from treatment. 33 34

Practically all the included studies in this review assessed direct costs only. In this regard, leaving the indirect costs unexplored confers a partial assessment of reality, since the latter can lead to even more economical burden than the former. 29 Future studies should include total costs, to better approximate the real costs and benefits of each therapy. Furthermore, a recent review underscores the lack of standardization in reporting costs, noting that they vary widely based on location and date. 35 As DBS technology rapidly evolves, we are already seeing significant changes, such as the implementation of remote adjustments via telemedicine and the development of “closed loop” systems.

In Latin America, there are no published studies on this topic. A recent publication assessed costs of PD therapy in Brazil, showing an average annual cost of $4,020.47 per capita, from which 49% of it is related to out-of-pocket expenditures. 36 Economic evaluations assessing costs and benefits from medical versus surgical therapy among PD subjects is an unmet need in Latin America. Better governmental policies for health services will be created as more information is available from its own population.

The limitations of the present study lie in the heterogeneity of its population. Most published studies were performed in high-income countries, with no information regarding Africa and Latin America. Differences in data processing (Markov, semi-Markov, and either prospective or retrospective studies), country, currency type and value, and year in which the analysis was performed, were the main sources of heterogeneity. Social network analysis depicted that correlations between ICER/ICUR and outcomes are highly variable, and outcomes are not uniform due to the studies' time and different employed utilities.

In conclusion, DBS has been widely accepted as an adjuvant therapy for patients with PD complications that cannot be controlled by pharmacological therapy. Moreover, surgical therapy with DBS will not only ameliorate motor and nonmotor symptoms but also diminish pharmacological adverse events, as drugs doses can be reduced or even suppressed. Further economic evaluations assessing either cost-effectiveness or cost-utility between medical and surgical treatments in developing countries are needed. Decision making policies will change over time as more information is obtained from this type of study.
